# CCN2/CTGF promotes liver fibrosis through crosstalk with the Slit2/Robo signaling

**DOI:** 10.1007/s12079-022-00713-y

**Published:** 2022-12-05

**Authors:** Liya Pi, Chunbao Sun, Natacha Jn-Simon, Sreenivasulu Basha, Haven Thomas, Victoria Figueroa, Ali Zarrinpar, Qi Cao, Bryon Petersen

**Affiliations:** 1 Department of Pathology, Tulane University, New Orleans, LA USA; 2 Department of Diagnostic Radiology and Nuclear Medicine, University of Maryland School of Medicine, Baltimore, MD USA; 3 Department of Surgery, Gainesville, USA; 4 Department of Pediatrics, University of Florida, Gainesville, FL USA

**Keywords:** Cellular communication network factor 2 (CCN2)/Connective tissue growth factor (CTGF), Slit2, Robo1, Carbon tetrachloride, Liver fibrosis, Hepatic stellate cells, Hepatocellular carcinoma

## Abstract

Liver fibrosis is the common outcome of many chronic liver diseases, resulting from altered cell-cell and cell-matrix interactions that promote hepatic stellate cell (HSC) activation and excessive matrix production. This study aimed to investigate functions of cellular communication network factor 2 (CCN2)/Connective tissue growth factor (CTGF), an extracellular signaling modulator of the CYR61/CTGF/Nov (CCN) family, in liver fibrosis. Tamoxifen-inducible conditional knockouts in mice and hepatocyte-specific deletion of this gene in rats were generated using the Cre-lox system. These animals were subjected to peri-central hepatocyte damage caused by carbon tetrachloride. Potential crosstalk of this molecule with a new profibrotic pathway mediated by the Slit2 ligand and Roundabout (Robo) receptors was also examined. We found that *Ccn2/Ctgf* was highly upregulated in periportal hepatocytes during carbon tetrachloride-induced hepatocyte damage, liver fibrosis and cirrhosis in mice and rats. Overexpression of this molecule was observed in human hepatocellular carcinoma (HCC) that were surrounded with fibrotic cords. Deletion of the *Ccn2/Ctgf* gene significantly reduced expression of fibrosis-related genes including *Slit2, a smooth muscle actin (SMA)* and *Collagen type I* during carbon tetrachloride-induced liver fibrosis in mice and rats. In addition, Ccn2/Ctgf and its truncated mutant carrying the first three domains were able to interact with the 7th -9th epidermal growth factor (EGF) repeats and the C-terminal cysteine knot (CT) motif of Slit2 protein in cultured HSC and fibrotic murine livers. Ectopic expression of Ccn2/Ctgf protein upregulated Slit2, promoted HSC activation, and potentiated fibrotic responses following chronic intoxication by carbon tetrachloride. Moreover, Ccn2/Ctgf and Slit2 synergistically enhanced activation of phosphatidylinositol 3-kinase (PI3K) and AKT in primary HSC, whereas soluble Robo1-Fc chimera protein could inhibit these activities. These observations demonstrate conserved cross-species functions of Ccn2/Ctgf protein in rodent livers. This protein can be induced in hepatocytes and contribute to liver fibrosis. Its novel connection with the Slit2/Robo signaling may have therapeutic implications against fibrosis in chronic liver disease.

**Supplementary information:**

The online version contains supplementary material available at 10.1007/s12079-022-00713-y.

## Introduction

Hepatic wound healing as a normal biological process involves inflammation, proliferation, and extracellular matrix (ECM) remodeling after liver injury. This process must be coordinated through cell-cell and cell-matrix interaction to ensure efficient communication of internal and external signaling. However, imbalanced signaling occurs during chronic liver injury. As a result, persistent damage causes profibrotic signaling leading to hepatic stellate cell (HSC) activation and excessive ECM deposition in liver fibrosis. Scar tissues disrupt normal blood flow and sequester hepatocytes in regenerative nodules that eventually result in portal hypertension, cirrhosis, and even liver cancer development. Identification of the disturbed signaling in cell-cell and cell-matrix interactions would facilitate the development of preventive and therapeutic approaches for liver fibrosis and possibly for hepatocellular carcinoma (HCC) (Kisseleva and Brenner 2021).

The Slit family of secreted proteins (Slit1, 2, and 3) were originally discovered as neuronal guidance cues that bind to Roundabout receptors (Robo1, 2, 3, and 4) in the immunoglobulin (Ig) superfamily. Activation of the Slit2-Robo1 signaling is critically involved in liver fibrosis by activating HSCs (Chang et al. 2015). Moreover, Slit2 can act through Robo2 leading to modulation of the fibrogenic activity and migration of HSC (Zeng et al. 2018). Activation of PI3K and ERK signaling is involved in initiating HSC activation, whereas blocking the PI3K/AKT signaling can decrease expression of fibrogenesis-related genes in activated HSCs during liver fibrogenesis (Son et al. 2009), (Munoz-Felix et al. 2016). Slit2 has been shown to significantly increase the expression of pro-fibrotic mediators including Cellular communication network protein 2/Connective tissue growth factor (Ccn2/Ctgf) and Collagen through activation of PI3K/AKT pathway (Zeng et al. 2018). Therefore, Slit2 and its receptors promote liver fibrogenesis with a direct effect on HSCs.

Ccn2/Ctgf is a matricellular protein (Perbal, Tweedie, and Bruford 2018) and is commonly co-expressed with transforming growth factor (TGF)β in a diverse variety of fibrotic disorders. This protein modulates cell adhesion, migration, differentiation, and apoptosis through binding to growth factors, ECM proteins, and cell surface receptors (Gressner and Gressner 2008). It contains a four modular protein structure and elicits adhesive signaling by acting as a linker between ECM and HSC. Moreover, it can potentiate the profibrogenic action of TGFβ, and control activation of HSCs and ductular reaction during liver damage (Huang and Brigstock 2012; Pi et al. 2008; Pi et al. 2015). Overexpression of CCN2/CTGF has been found in fibrotic diseases involving many organ systems (Gressner and Gressner 2008). Inhibition of CCN2/CTGF using pharmacological to targeted siRNA approaches have been shown to reduce fibrosis in a variety of experimental models (George and Tsutsumi 2007; Brigstock 2009; Li et al. 2008; Li et al. 2006; Lipson et al. 2012). Clinical trials targeting CCN2/CTGF for anti-fibrosis treatment are ongoing (Richeldi et al. 2020; Raghu et al. 2016). In this paper, we conditionally deleted the *Ccn2/Ctgf* gene in both mice and rats, and asked whether loss of this gene influenced liver fibrosis. We have identified Ccn2/Ctgf as an interactor for multiple angiogenic regulators including Slit ligands (Pi et al. 2012). Herein, we characterized the interaction between Ccn2/Ctgf and Slit2. This interaction was associated with enhanced Slit2/Robo signaling, HSC activation, and liver fibrosis.

## Materials and methods

### Animal experiments

Wild type C57BL6 mice (8–10-week-old) were subjected to a single dose of CCl_4_ (1 µl/g body weight) prediluted 1:3 in olive oil through intraperitoneal injection (IP) for induction of acute liver injury. In addition, *Ccn2/Ctgf* conditional mouse knockouts were generated through tamoxifen administration of floxed homozygotes (obtained from Dr. Andrew Leask in College of Dentistry, University of Saskatchewan) carrying the human ubiquitin C promoter driven Cre transgene that was fused to a triple mutant form of the human estrogen receptor (*ubc-Cre/ERT2*) according to our previous report (Pi et al. 2015). Liver fibrosis was induced through IP injection of CCl_4_ (0.5 µl/g body weight) twice a week for six weeks the *Ccn2/Ctgf* conditional mouse knockouts.

The hepatocyte-specific *Ccn2/Ctgf* knockouts (KO) in rats were generated based on Cre-lox system as follows. At first, rat *Ccn2/Ctgf floxed homozygotes* (designed as *Ccn2/Ctgf*^*flox/flox*^) were obtained from SMOC INC (Shanghai, China) based on fee-for service. To generate the animals, the clusters of regularly interspaced short palindromic repeats (CRISPR) technology were used to insert two loxP cassettes on 5’ and 3’ untranslated region (UTR) of the *Ccn2/Ctgf* gene (ENSRNOG00000015036). As shown in Supplemental Fig. 1, one loxP site was designed in a target donor construct that contained DNA sequences for a 5’ homologous arm using *Cas9* mRNA and two guide RNA (gRNA)s (gRNA1: 5’ GCTGAAGAGGCAGATACCAC GGG 3’; gRNA2: 5’ AGCTGAAGAGGCAGATACCA CGG 3’). The other loxP site was designed in the target donor construct that contained DNA sequences for a 3’ homologous arm using gRNA3 (5’ CAGTGACAGAACGCACACTA AGG 3’) and gRNA4 (5’ GCACACTAAGGTGAGCCTCC TGG 3’). The donor vector was constructed by in-fusion technology, and contained the 5’ homologous arm, a flox region, and the 3’ homologous arm. The mixtures of Cas9 mRNA, gRNAs and donor vector were microinjected into fertilized eggs of Sprague Dawley (SD) rats. The F0 rats were identified by PCR based genotyping and crossed with wildtype SD to generate F1 rats. By long-PCR identification and sequencing, we confirmed there were 7 F1 rats carrying the targeted allele (*Ccn2/Ctgf*^*flox*^). Homozygotes were generated by crossing female and male F1 rats. For genotyping the offspring, short-PCR by P1 and P2 primer pair were used to identify homozygous, heterozygous and wildtype rats (Supplemental Fig. 1). For generation of the hepatocyte-specific knockouts, *Ccn2/Ctgf*^*flox/flox*^ rats (10-week-old age) received tail vein injection of recombinant adeno-associated virus serotype 8 (AAV8) virus expressing codon-improved Cre recombinase (iCre) under the control of the human thyroxine-binding globulin (TBG) promoter (AAV8-Cre). The virus was purchased from Vector Biolabs (Malvern, PA) and was given at 2 × 10^12^ plaque forming unit (pfu) per rat. The same amount of AAV8-green fluorescence protein (GFP) was injected into animals as a control. Three weeks after the viral administration, the AAV8-Cre infected knockouts (KO) and AAV8-GFP-infected controls (CT) were exposed to 6-week CCl_4_ (1 µl/g body weight) intoxication for liver fibrosis induction as described above. Oil was injected into both AAV8-GFP and AAV-Cre-treated rats in parallel studies. In addition, wild type SD rats (8-week-old age, 200–220 g) received IP injection of chronic CCl_4_ (1 µl/g body weight) up to 1.5 to 3 months for detection of expression pattern of Ccn2/Ctgf protein in fibrotic or cirrhotic livers.

**Fig. 1:**
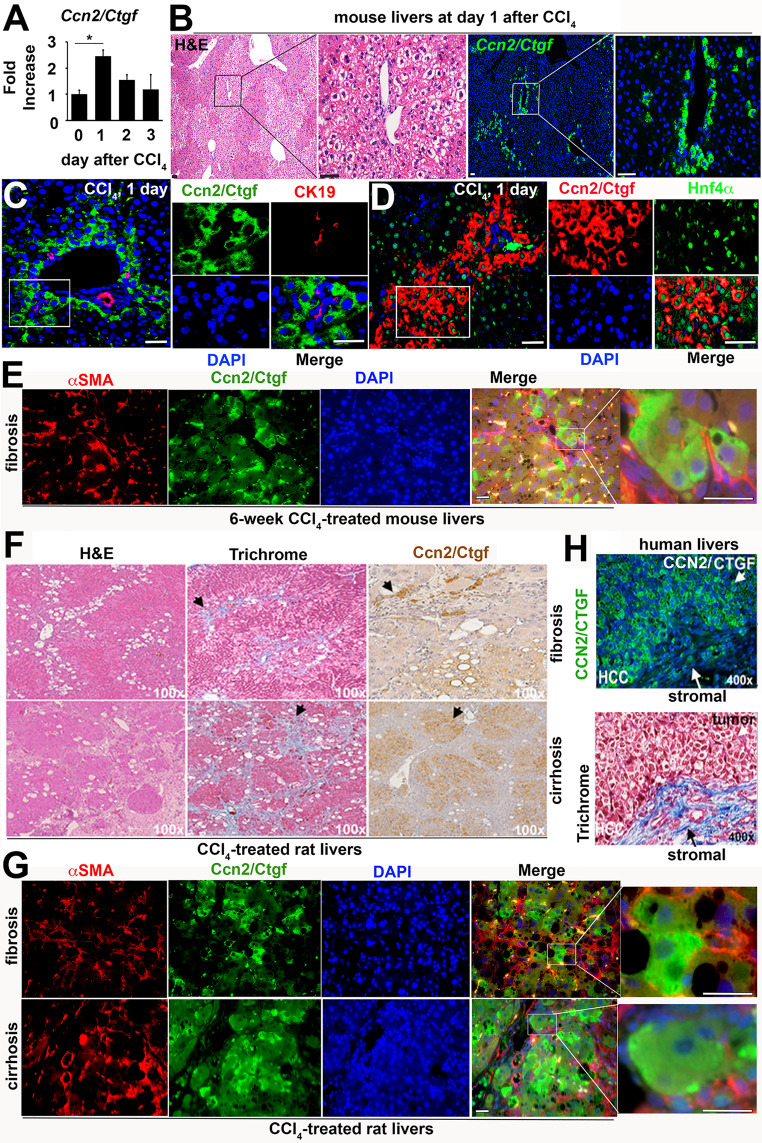
*Ccn2/Ctgf* is induced during hepatocyte damage, liver fibrosis and cirrhosis of rodent models as well as in human HCC. C57BL6 mice (n = 5) in (A-D) were subjected to IP injection of one dose of CCl_4_ (1ml/g body weight). (A) qRT-PCR analysis detected upregulation of *Ccn2/Ctgf* transcript at 24 h post CCl_4_ intoxication from 5 mice. Values are means ± SD from triplicate studies. *P < 0.05. (B) H&E and immunofluorescent staining detected Ccn2/Ctgf localization in periportal zones (PT) using consecutive slides. (C and D) The immunofluorescent staining labeled Ccn2/Ctgf expression in Hnf4a^+^ hepatocytes but not CK19^+^ cholangiocytes. (E) Dual staining for Ccn2/Ctgf and aSMA in fibrotic mouse livers (n = 3) that received 6-week CCl_4_ intoxication (0.5 ml/g body weight, twice/week). (F) Trichrome and Ccn2/Ctgf staining were performed in liver fibrosis or cirrhosis sections from Sprague Dawley (SD) rats (n = 3 per group) that were exposed to chronic CCl_4_ (twice a week, 0.5ml/g body weight) for 6 weeks or 3 months respectively. (G) Dual staining for Ccn2/Ctgf and aSMA was carried out on SD rats (n = 3 per group) that received chronic CCl_4_ for 6 weeks (upper panel) or 90 days (lower panel) respectively. (H) CCN2/CTGF and Trichrome staining were performed on consecutive sections of human HCC section (n = 2). Scale bar: 100 mm

Mice (8-week-old) that ectopically expressed a rat *Ccn2/Ctgf* gene with FLAG epitope at C termini were generated through AAV8 delivery systems (AAV8-Ccn2/Ctgf:FLAG). The expression vector pTR-UF12 is a gift from Dr. Alfred S Lewin at University of Florida Gainesville. It contains the 381–base pair (bp) cytomegalovirus enhancer immediate early gene enhancer and the 1352-bp chicken b actin promoter-exon1-intron1 (CBA) promoter (Qi et al. 2007). This plasmid is linked to GFP via a 637-bp poliovirus internal ribosomal entry site. The Ccn2/Ctgf:FLAG DNA fragment was amplified with a primer set 5′ GCTAGCCCTCCTGCCGCGCCCCGACCATGCTCGCC3′ and 5′ GGATCCCGCCATGTCTCCATACATCTTCCTGT3′ and cloned into flag-pGEM-vector according to our previous report (Pi et al. 2005). The resulting cDNA and FLAG sequences were further cloned into *SpeI* site of the pTR-UF12 vector and expressed under the control of CBA promoter. Recombinant AAV8 virus was packaged via fee for service in the Powell Gene Therapy center at the University of Florida Gainesville. Animals received 5 × 10^11^ AAV8-Ccn2/Ctgf:FLAG through tail vein injection. AAV8-GFP control was injected in parallel studies. Six-week intoxication by CCl_4_ (0.5 µl/g body weight) was introduced to induce liver fibrosis in infected mice at three weeks later after the viral administration.

All animals in this paper were euthanized to assess the extent of liver fibrosis at two days after the last dose of chronic CCl_4_ intoxication. Isoflurane overdose followed by cervical dislocation was used for animal euthanasia. Liver tissues were harvested for histological, molecular, and biochemical analyses. All animal protocols were approved by the Animal Care and Usage Committee at Tulane University and were conducted in compliance with their guidelines.

### Histological analysis

Human HCC sections were obtained from BioChain (Newark, CA). Mouse and rat livers were fixed in 4% paraformaldehyde. Histological analyses were performed with standard protocols using OCT or paraffin-embedded Sect. (6 μm). Primary antibodies used were rabbit anti-hepatocyte nuclear factor (Hnf)4a (Santa Cruz biotechnologies, Dallas, TX, USA), rabbit anti-Ccn2/Ctgf (Abcam, Cambridge, United Kingdom), and rabbit anti-cytokeratin (CK)19 (Abcam). Detection was carried out according to the manufacturer’s instructions using the ABC-Elite kit with ImmPACT DAB substrate (Vector Laboratories, Burlingame, CA, USA). Collagen deposition was measured by Sirius Red staining or Trichrome blue according to previous publication(Pi et al. 2015). Alexa Fluor® 596 conjugated a smooth muscle actin (SMA) antibody (Abcam) was also used for double staining with the Ccn2/Ctgf rabbit antibody (Abcam). For estimation of stained positive areas, images were captured with CellSens software using an Olympus BX 51 upright fluorescence microscope outfitted with an Olympus DP80 camera, Plan Fluorite objectives and a LED transmitted light source (Olympus, Waltham, MA, USA). DAB stained areas were quantified from 10 random fields of images using Image J software (http://rsb.info.nih.gov/ij/) and IHC profiler according to published methods (Varghese et al. 2014).

### Cell culture, immunofluorescent staining, and fluorescent microscopy

Primary hepatocytes were isolated from *Ccn2/Ctgf*^*f/f*^ rats and cultured in William’s E media according to our previous publication (Pi et al. 2008). In brief, rats were IP injected with chronic CCl_4_ (0.5 ml/g body weight, twice a week for 6 weeks). Two days after the last injection, livers were perfused by the two-step Collagenase digestion method (Pi et al. 2015). Ca^2+^ free Hanks buffer containing EGTA (34 mg/ml, Sigma-Aldrich, St. Louis, MO, USA) and 0.5% BSA were used in the first step of perfusion. Then enzymatic digestion was carried out in Hanks buffer containing 0.05% (w/v) Collagenase II (Worthington Biochemical Corporation, Lakewood, NJ, USA) and CaCl_2_ (58.8 mg/ml) via recirculation. The digested livers were further disassociated in the Hanks buffer. Hepatocytes were isolated after centrifuge at 50 x and 45% Percoll followed by 200 x g centrifuge. The cell pellet was re-suspended in complete William’s E medium (Thermo Fisher Scientific, Waltham, MA, USA) containing L-glutamine (2mM), 5% fetal bovine serum (FBS) (Invitrogen, Waltham, MA, USA), insulin (100 nM), dexamethasone (100 nM), penicillin (100U/ml) and streptomycin (100 mg/ml). Purity of the isolated hepatocytes was about 85% according to staining of the hepatocyte marker Albumin using a rabbit antibody (Invitrogen) as shown in Supplemental Fig. 2. The isolated cells were seeded in Collagen coated plates with complete Williams’ E media at 37^o^C with 5% CO_2_. Four hours later, new complete Williams’ E media were added. The cultured *Ccn2/Ctgf*^*f/f*^ hepatocytes were transduced with AAV8-Cre at a multiplicity of infection (MOI) of 100,000 viral genomes/cell to delete the *Ccn2/Ctgf* gene in vitro. The same amounts of AAV8-GFP were transduced in parallel wells as control groups. GFP visualization indicated nearly 100% transduction rate (Supplemental Fig. 3). Three days later, the infected cells were used for immunostaining or Western analysis. Double staining was performed using the rabbit anti-Albumin and a mouse anti-Ccn2/Ctgf antibody (Santa Cruz Biotechnologies) followed by detection with Alexa Fluor® 488 or 596 conjugated Donkey secondary antibodies.

Primary HSCs were isolated from wildtype mice according to our previous publication(Pi et al. 2015). They were maintained in Dulbecco’s Modified Eagle’s Medium (Invitrogen) supplemented with 10% FBS and antibiotics at 37 °C in a 5% CO_2_ humidified atmosphere. Double staining was performed using a rabbit anti-Slit2 (Proteintech, Rosemont, IL, USA) and the mouse anti-Ccn2/Ctgf antibody (Santa Cruz Biotechnologies).

Recombinant mouse Slit2 protein (R&D Biosystems, Minneapolis, MN, USA), recombinant mouse Ccn2/Ctgf protein (Abcam), or human ROBO1-Fc chimera protein (R&D Biosystems), phosphate buffered saline (PBS) vehicle, or in combination were added to primary mouse HSC cells that were pre-starved for 16 h. 30 min after the stimulation, the cells were lysed for total protein extraction followed by Western blotting for the serine/threonine kinase AKT, p-AKT, phosphatidylinositol 3-kinase (PI3K), and p-PI3K. Levels of aSMA and actin were examined after 24-hour stimulation by Western blotting.

### RNA isolation and reverse transcriptase-polymerase chain reaction (RT-PCR)

Total RNA isolation, RT-PCR, and quantitative RT-PCR (qRT-PCR) analyses were performed in the same conditions according to our previous publication (Zhou et al. 2020). Primers included: 5’ TGGCGAGATCATGAAAAAGAA 3’ (forward) and 5’ GCCTTTGTCATCGTCATCCT 3’ (reverse) for for rat *Ccn2/Ctgf:FLAG*; 5’ ATGCTCGCCTCCGTCGCGGGT 3’ (forward) and 5’ GCCTTGTCTCCATACATCTTCCTG 3’ (reverse) for the full-length *rat Ccn2/Ctgf*; 5’ GGCAGACACTGTCCCTATCG 3’ (forward) and 5’ ATCTATCTTCGTGATCCTCGTGA 3’ (reverse) for mouse *Slit2*; 5’ GTGCGTCTGGTGTGAATGAG 3’ (forward) and 5’ ATGCTCCTGGTGCAACTGTG 3’ (reverse) for rat *Slit2*; 5’ GTGCAAGCTGAACAACAGGA 3’ (forward) and 5’ CCAGCATCCACATTCTCCTT 3’ (reverse) for *iCre*; 5’ TTACTTGTACAGCTCGTCCATG 3’ (forward) and 5’ GCCACCATGGTGAGCAAGGG 3’ (reverse) for *GFP*. Other primers were previously reported (Pi et al. 2015), (Zhou et al. 2020). All qRT-PCR experiments were performed in triplicate using cDNA samples from independent RNA sets and the relative amount of target mRNA was calculated using the delta‐delta CT method according to published reports (Livak and Schmittgen 2001) and was normalized against reference gene (*18 S*) in each sample.

### Yeast two-hybrid analyses

The cDNAs of rat Ccn2/Ctgf (Genbank accession# NM_022266), and its mutants were cloned into *NotI* and *SalI* sites in pPC97 vector containing DNA binding domain (BD) of the *GAL4* gene as described in our previous report (Pi et al. 2012). The cDNA fragments for human SLIT2 (GenBank#: AF133270.1) were cloned into *NotI* and *SalI* sites in pPC86 vector containing GAL4 Activation domain (AD). Primers for the BD fused C terminal fragment of SLIT2 (BD:SLIT2C) were 5’ GAGGTCGACCGACTTCCAGAAGGTG 3’ (P1) and 5’ TAGCGGCCGC AGGACACACACCTCG 3’ (P2). The BD fused C terminal cysteine knot of SLIT2 (BD:SLIT2-CT) were amplified using 5’ GAGGTCGACCAGGCTTTCAGGTCTG 3’ and P2 primer. The BD fused SLIT2 carrying the epidermal growth factor (EGF) repeats 7–9 (BD:SLIT2-EGF) were amplified using P1 primer and 5’ TAGCGGCCGCTGCTTTTGGTAATAAT 3’. Yeast two-hybrid analyses were performed according to our previous publications (Pi et al. 2008; Pi et al. 2012). BD constructs contained tryptophan gene and AD constructs expressed leucine gene. Therefore, the co-transformants that carried out BD and AD constructs could grow in the Synthetic Complete (SC) agar media lacking tryptophan and leucine (Clontech Laboratories Inc, Mountain View, CA, USA). *HIS3* reporter gene was designed in the yeast two-hybrid system to detect interaction between proteins that were fused to GAL4 BD or AD in vectors. 3-aminotriazole (3-AT) (Sigma) was added in SC media to eliminate the transcriptional background mediated by *HIS3* reporter gene. Only interactors could grow in SC agar media containing 3-AT but lacking tryptophan, leucine, and histidine.

### Protein expression, purification, and immunoprecipitation

The full-length human SLIT2 cDNA (Genbank accession# BC117190) was obtained from Open Biosystems (Huntsville, AL, USA) and cloned to fuse with the MYC epitope at its 3’ end in pSEC-B vector at restriction enzyme sites *KpnI* and *XhoI*. The resulting SLIT2:MYC construct, together with the 3xFLAG tagged Ccn2/Ctgf or mutants, was transfected into Chinese hamster ovary (CHO) cells using lipofectamine 3000 (Invitrogen). Immunoprecipitation assays were performed at two days after cell transfection. Conditioned media from the transfected cells were adjusted to contain 1× Tris-buffered saline (TBS; 20 mM Tris-HCl, pH 7.4, and 150 mM NaCl) and proteinase inhibitor cocktail (Sigma-Aldrich). For co-immunoprecipitation assays in AAV8-Ccn2/Ctgf:FLAG- or AAV8-GFP-infected murine livers, total protein homogenates were also extracted using 1x TBS buffer supplemented with 0.5% Triton-100 and proteinase inhibitor cocktail. The conditioned media, or cell lysates were incubated with M2 antibody-conjugated agarose (Sigma-Aldrich) for 16 h. After extensive wash with TBS buffer, the bound proteins were eluted with 100 mM glycine (pH 2.5), separated in 6% SDS-PAGE gel, and immunoblotted with the rabbit Slit2 antibody (Proteintech).

For preparation of maltose-binding protein (MBP) fusion proteins, the C terminal regions of SLIT2 (SLIT2C) tested were removed from the pPC86 yeast two-hybrid construct and inserted at *Sal*I and *Not*I sites before the 3′ of MBP in a modified pMAL vector described previously (Pi et al. 2012). Fusion proteins were induced in *Escherichia coli (E. coli)* DE3 strain with 0.4 mM isopropyl-β-d-thiogalactopyranoside, purified using amylose beads (New England Biolabs, Ipswich, MA, USA) with column buffer containing 20 mM Tris-HCl (pH 7.4), 200 mM NaCl, and 1 mM EDTA, and eluted with 10 mM maltose.

### Solid-phase protein-binding assays

Solid-phase protein-binding assays were carried out as follows. Microplates were coated with purified MBP or MBP-SLIT2C proteins with concentrations about 10, 50, 100, or 150 nM at 4 ^o^C overnight. Then the wells were washed with TBS-T buffer (TBS with 0.05% Tween 20) followed by blocking in TBS-T containing 1% BSA for two hours at room temperature. The wells were incubated with purified recombinant rat Ccn2/Ctgf:3xFLAG protein (1 mg/ml) for 2 h at room temperature. Unbound protein was removed by extensive washing with TBS-T. M2 antibody-conjugated HRP (Sigma) was prepared at 1:20,000 dilution and incubated with the wells for 1 h at room temperature. Tetramethylbenzidine was added as the substrate (R&D Systems), and the reaction was stop by 1 M H_2_SO_4_. The absorbance at 450 nm of each well was measured with an EnVision 2104 multimode microplate reader (PerkinElmer, Hopkinton, MA, USA).

### Western blotting

Total proteins were extracted from mouse livers or cultured cells in RIPA buffer containing proteinase inhibitors (Sigma). Total protein lysates (50 µg) were boiled in 1x Laemmli buffer containing 5% β-mercaptoethanol, separated in SDS-PAGE gel, and electro-transferred onto polyvinylidene difluoride membrane for immunoblotting. Primary antibodies included rabbit anti-Ccn2/Ctgf (Abcam), rabbit anti-Slit2 (Proteintech), rabbit anti-aSMA (Proteintech), rabbit anti-Collagen (Proteintech), rabbit anti-p-AKT (Cell Signaling Technology, Danvers, MA, USA), rabbit anti-AKT (Cell signaling), rabbit anti-p-PI3K (Cell signaling), rabbit anti-PI3K (Cell signaling), and rabbit anti-GAPDH (Abcam). Detection was carried out using horseradish peroxidase-conjugated secondary antibodies (Santa Cruz biotechnologies) and the ECL Plus kit (Amersham Biosciences, Piscataway, NJ, USA).

### Statistical analysis

GraphPad Prism 6.0 (GraphPad Software, San Diego, CA, USA) was used for statistical analysis. Statistical significance (*P* < 0.05) was evaluated using the unpaired *t-*test and one-way analysis of variance (ANOVA).

## Results

### Upregulation of *Ccn2/Ctgf* gene during CCl_4_-induced hepatocyte damage, liver fibrosis, cirrhosis, and human HCC

CCl_4_ is a potent hepatotoxin known to cause oxidative stress and pericentral damage leading to steatohepatitis, fibrosis, cirrhosis, and liver cancer in experimental models. We utilized this chemical and observed upregulation of *Ccn2/Ctgf* transcript within 24 h post one acute dose of CCl_4_ in mice (Fig. 1A). This observation was consistent with our previous publication about upregulation of Ccn2/Ctgf during CCl_4_-induced acute damage in absence or presence of moderate ethanol pre-exposure (Zhou et al. 2020). Hematoxylin and eosin (H&E) and immunofluorescent staining for Ccn2/Ctgf on consecutive sections indicated that this protein was localized in periportal zones of the CCl_4_-treated murine livers (Fig. 1B), although there was little staining of this molecule in untreated livers at day 0 (Supplemental Fig. 4). Additional immunofluorescent staining for the biliary specific marker CK19 confirmed that Ccn2/Ctgf immunoreactive cells were located within periportal regions but not in cholangiocytes (Fig. 1C). Moreover, these Ccn2/Ctgf highly expressed cells were stained positive for the hepatocyte marker Hnf4a (Fig. 1D). We also stained fibrotic murine livers that were damaged through 6-week chronic CCl_4_ intoxication. Dual staining showed that these Hnf4a^+^ hepatocytes were closely associated with activated aSMA^+^ HSC (Fig. 1E). This observation agrees with our previous findings about lack of Ccn2/Ctgf promoter activity in aSMA^+^ or desmin^+^ myofibroblast cells during liver injury after CCl4 intoxication in mice (Pi et al. 2015).

In rats, abundant Ccn2/Ctgf protein was also found in cells proximal to macrovesicular regions, fibrotic zones, and cirrhotic nodules after chronic CCl_4_ administration as shown in Fig. 1 F. Dual staining detected close association of these ballooning hepatocytes with activated aSMA^+^ HSC (Fig. 1G and Supplemental Fig. 5), implicating paracrine effects of hepatocyte-derived Ccn2/Ctgf on HSC during liver fibrosis. CCN2/CTGF overexpression was also found in HCC tumor cells that were surrounded by heavily fibrotic stroma (Fig. 1H). Taken together, these results indicated cross-species upregulation of *Ccn2/Ctgf* gene during hepatocyte damage, liver fibrosis, cirrhosis, and HCC development.

### Systemic deletion of *Ccn2/Ctgf* gene reduces myofibroblast activation and liver fibrosis in adult mice after chronic CCl_4_ intoxication

**Fig. 2:**
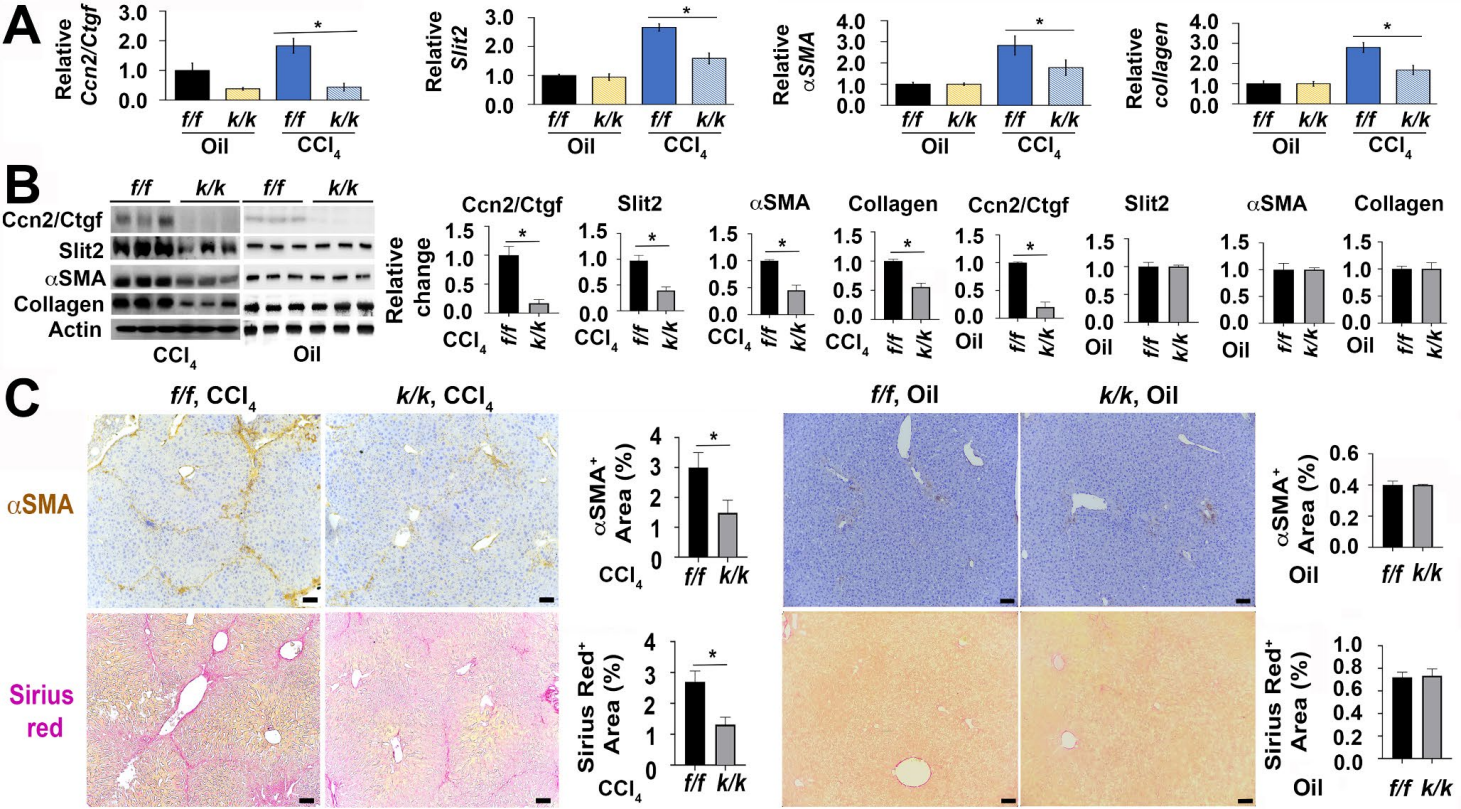
*Ccn2/Ctgf* conditional knockouts exhibited reduced myofibroblast activation and liver fibrosis as evidenced by low production of Slit2, aSMA and Collagen type I at mRNA and protein levels in adult mice after chronic CCl_4_ intoxication. The conditional knockout mice (*Ccn2/Ctgf*^*k/k*^, n = 3) and floxed littermates (*Ccn2/Ctgf*^*f/f*^, n = 3) were subjected to chronic intoxication by IP injection of CCl_4_ (0.5ul/ug body weight, twice a week) for 6 weeks. Olive oil was injected in additional experiments (n = 3 per group). (A) Downregulated transcripts of *Slit2, aSMA* and *Collagen type I* genes were found in the CCl_4_-treated *Ccn2/Ctgf*^*k/k*^ livers by qRT-PCR analysis. In contrast, no difference was found compared to additional oil-treated groups. Values represent means ± SD in relation to oil treated *Ccn2/Ctgf*^*f/f*^ controls from three independent experiments. *P < 0.05. (B) Lower levels of these gene products were also detected compared to controls in Western analysis. Graphs are from densitometric analyses based on band intensity. Data are expressed as means ± SD in relation to *Ccn2/Ctgf*^*f/f*^ controls in corresponding CCl_4_ and oil treated *Ccn2/Ctgf*^*k/k*^ groups from three different studies. *P < 0.05. (C) Reduced levels of aSMA and Collagen were found in IHC and Sirius Red staining of the CCl_4_-treated *Ccn2/Ctgf*^*k/k*^ livers in comparison to the CCl_4_-treated *Ccn2/Ctgf*^*f/f*^ controls. Values were means ± SEM based on quantification of images from more than 10 fields per mouse (n = 3 mice per group). *P < 0.05. Scale bar: 500 mm in (C)

To determine the importance of *Ccn2/Ctgf* gene in CCl_4_-induced liver fibrosis, we deleted exon 4 utilizing mice that were homozygous for the floxed-*Ccn2/Ctgf* allele and hemizygous for the *ubc-Cre/Ert2* transgene according to our previous report (Pi et al. 2015). For simplicity, the conditional knockouts were termed as *Ccn2/Ctgf*^*k/k*^ in this study. Exon 4 deletion was generated after tamoxifen administration at one month before CCl_4_ administration. We compared the fibrogenic response in *Ccn2/Ctgf*^*k/k*^ knockouts with controls that were floxed homozygotes (*Ccn2/Ctgf*^*f/f*^) after a 6-week CCl_4_ administration. As shown in Fig. 2A, the CCl_4_-treated conditional knockout mice had deletion of the *Ccn2/Ctgf* gene and showed downregulation of *Slit2*, *aSMA* and *Collagen type I* transcripts as detected by qRT-PCR and Western blotting analyses (Fig. 2A and B). IHC revealed much smaller areas of fibrotic lesions detected in aSMA staining as well as Sirius red staining compared with *Ccn2/Ctgf*^*f/f*^ controls (Fig. 2C). In contrast, *Ccn2/Ctgf*^*k/k*^ and *Ccn2/Ctgf*^*f/f*^ did not exhibit any fibrotic responses in oil-treated conditions (Fig. 2A-C). These results indicated that *Ccn2/Ctgf* deficiency reduced CCl_4_-induced liver fibrosis in mice.

### The hepatocyte-specific deletion of *Ccn2/Ctgf*gene deceases myofibroblast cell activation and Collagen deposition in rats after chronic CCl_4_ intoxication

**Fig. 3:**
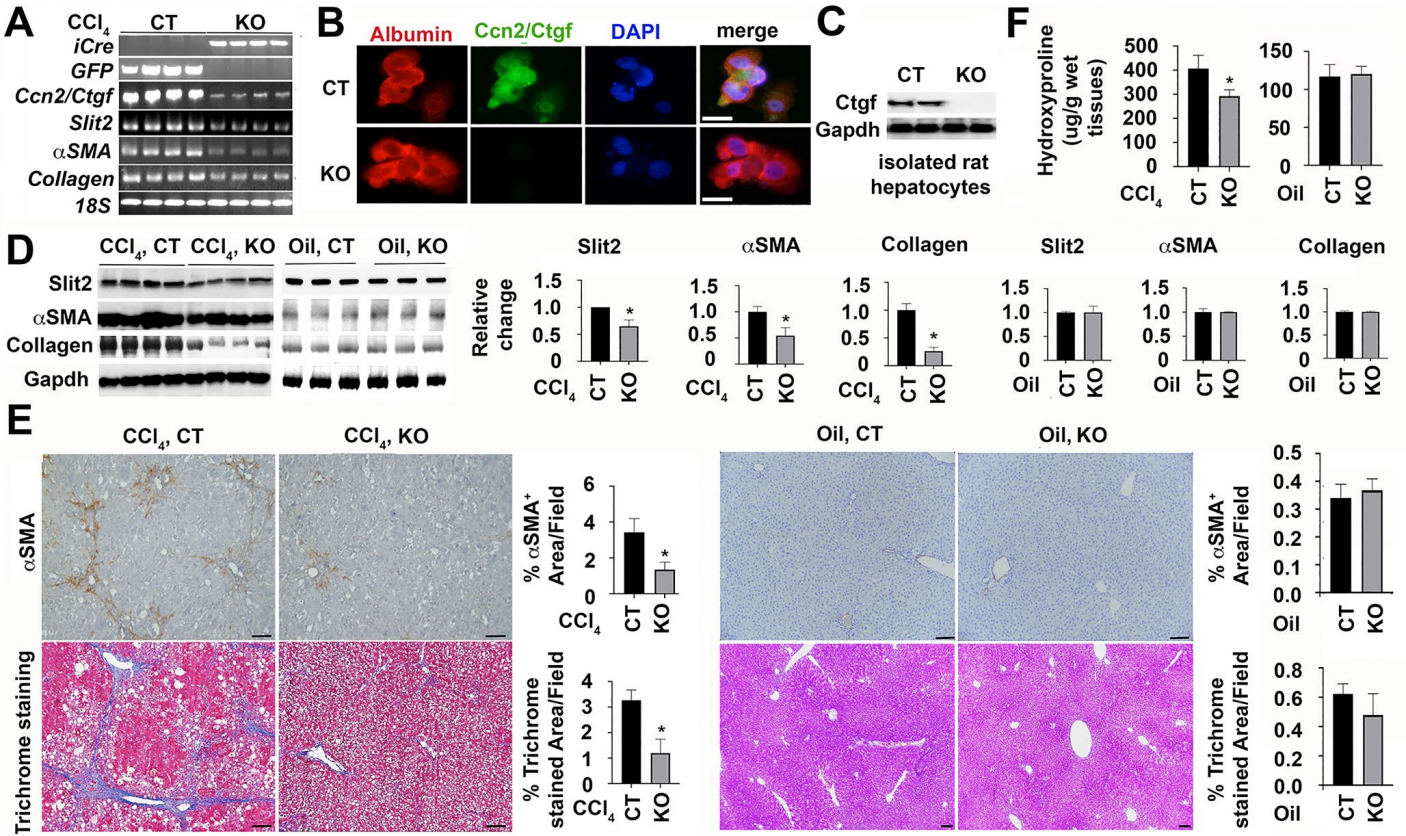
The hepatocyte-specific deletion of *Ccn2/Ctgf* gene is associated with low production of *Slit2*, *aSMA*, and *Collagen type I* genes during CCl_4_-induced liver fibrosis in rats. The hepatocyte-specific rat knockouts (KO, n = 4) were generated after tail vein injection of AAV8-iCre or AAV8-GFP as control (CT). These AAV8 infected livers received CCl_4_ intoxication for six weeks. Oil was also injected in parallel experiments. (A)The presence of iCre, decrease of the full-length *Ccn2/Ctgf*, and downregulation of *Slit2, aSMA* and *Collagen type I* genes were found in the KO livers by semi qRT-PCR analyses after chronic CCl_4_ intoxication. Graphs show relative changes that were expressed as means ± SD in relation to CT groups in corresponding CCl_4_ and oil-treated conditions (n = 3–4 per group). *P < 0.05. (B and C) Loss of Ccn2/Ctgf protein in albumin^+^ primary KO hepatocytes were confirmed by double staining (B) and Western blotting from total protein extracts of two different wells of the cultured primary KO hepatocytes (C). (D) Lower levels of aSMA and Collagen type I proteins were found in the KO rat livers than CT controls in Western blotting. Graphs indicate relative changes that were quantified in densiometric analyses. Values represent means ± SD in relation to CT groups in corresponding CCl_4_ and oil-treated conditions (n = 3–4 per group). *P < 0.05. (E) IHC and Sirius red staining detected lower levels of aSMA and Collagen in KO compared to CT group (n = 4 per group) that received chronic CCl_4_. Values were means ± SEM based on quantification of images from more than 10 fields per animal. *P < 0.05. (F) Decreased Collagen deposition was observed in the rat KO (n = 4) after measurement of hepatic hydroxyproline contents. Values were calculated as mg/g wet tested livers and represented means ± SD (n = 3–4 per group). *P < 0.05. Scale bar: 30 mm in (B) and 500 mm in (E)

To understand whether *Ccn2/Ctgf* has conserved functions in other species, we utilized the CRISPR technology and generated a novel line in which loxP sites were inserted before 5’ UTR or after 3’ UTR in the rat *Ccn2/Ctgf* gene (Supplemental Fig. 1A and B). Homozygotes for the floxed-*Ccn2/Ctgf* transgene were identified based on PCR-based genotyping (Supplemental Fig. 1C). Homozygous littermates received tail vein injection of AAV8-Cre or control AAV8-GFP virus followed by 6-week chronic exposure to CCl_4_. As shown in Fig. 3A, presence of iCre induced *Ccn2/Ctgf* deletion as evidenced by very low levels of the full-length *Ccn2/Ctgf* transcript in the AAV8-Cre infected KO livers in comparison to AAV8-GFP infected CT. Isolated primary hepatocytes exposed to AAV8-Cre were positive for albumin staining but had little immunoreactivity to Ccn2/Ctgf antibody (Fig. 3B). The loss of Ccn2/Ctgf in the KO hepatocytes was also verified in immunoblotting (Fig. 3C). This hepatocyte-specific loss of Ccn2/Ctgf affected liver fibrosis since there were significant decreases of *Slit2*, *aSMA* and *Collagen type I* genes at mRNA levels (Fig. 3A). Immunoblotting confirmed decreased levels of these fibrosis-related gene products in total liver homogenates of the CCl_4_-damaged KO (Fig. 3D). Decreased areas in aSMA staining and Trichrome staining were also observed in the KO (Fig. 3E). Determination of hepatic hydroxyproline content further demonstrated decreased deposition of Collagen in the damaged KO livers than controls (Fig. 3F). Additional oil-treatment in KO and CT mice did not cause any fibrotic responses (Fig. 3D-F). Taken together, these results indicated that loss of hepatocyte derived Ccn2/Ctgf in rat livers decreased fibrotic responses during injury following chronic CCl_4_ intoxication.

### Ccn2/Ctgf binds to Slit2 and potentiates Slit2/ROBO1 signaling in HSC in vitro

**Fig. 4:**
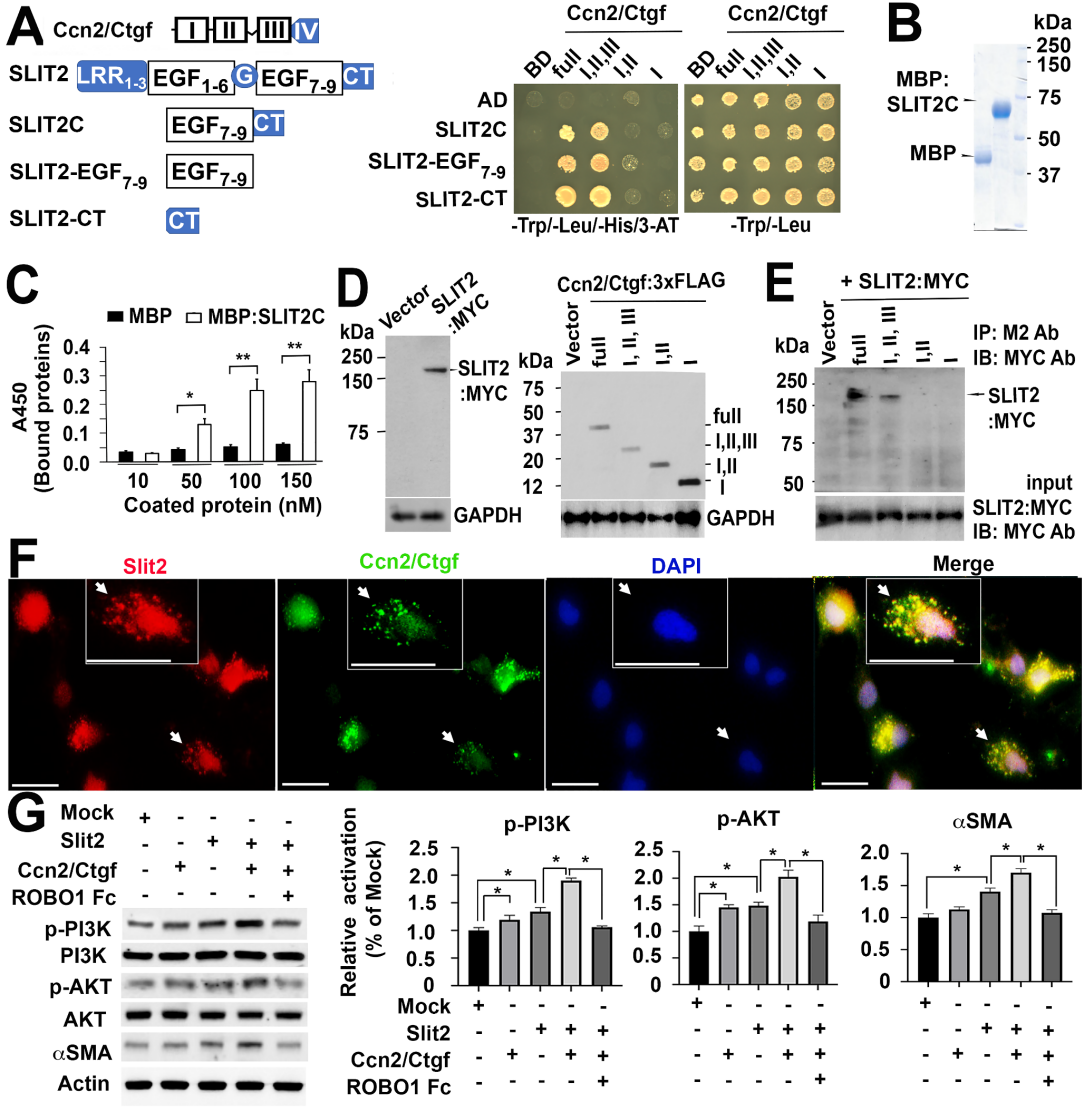
Ccn2/Ctgf binds to Slit2 and potentiates HSC activation in vitro. (A) Ccn2/Ctgf interacted with SLIT2-EGF_7 − 9_, SLIT2-CT and SLIT2C in yeast two-hybrid analyses. LRR: leucine rich repeat domain; G: laminin G-like module; CT: C-terminal cystein knot; AD: activation domain; BD: binding domain; Trp: tryptophan; Leu: leucine; His: histidine. (B) Purified MBP and MBP fused SLIT2C were resolved using SDS-PAGE gel electrophoresis and stained with Coomassie Brilliant Blue. (C) Dose-dependent bindings of Ccn2/Ctgf to MBP:SLIT2C in solid-phase assays. Data are presented as means ± SD in triplicate wells. *P < 0.05; **P < 0.01. (D) SLIT2:MYC, Ccn2/Ctgf, and truncated mutants were expressed in CHO cells. (E) SLIT2:MYC protein was specifically detected in complexes that were immunoprecipitated with M2 antibody (Ab) and 3xFLAG tagged Ccn2/Ctgf and Ccn2/Ctgf_I,II,III_. MYC Ab was used in immunoblotting. Equal input of SLIT2:MYC protein was added in the immunoprecipitation assays. (F) Co-localization of Slit2 and Ccn2/Ctgf proteins in primary mouse HSCs. Arrows in images and inserts indicate the same locations. Scale bar: 20 mm. (G) Western blot analyses showed that recombinant Ccn2/Ctgf protein potentiated Slit2-stimulated phosphorylation of PI3K and AKT as well as aSMA upregulation in cultured mouse HSCs, whereas presence of ROBO1-Fc chimera protein inhibited these actions mediated by Ccn2/Ctgf and Slit2. Values were means ± SD based on densitometric quantification of band intensities related to Mock controls in three independent experiments. *P < 0.05

Ccn/Ctgf protein contains a four-modular protein structure (Fig. 4A). Domain I is an insulin like growth factor-binding protein (IGFBP)1. Domain II is a von Willebrand type C (vWC) module. Domain III is a thrombospondin type-1 repeat (TSP1). Domain IV is a C terminal cysteine knot (CT) motif. Our previous studies have identified Ccn2/Ctgf as a binding protein to multiple angiogenic regulators including Slit3 after screening a yeast two-hybrid library that was generated from rat livers with oval cell activation induced by partial hepatectomy and 2-acetylaminofluorene (2-AAF) (Pi et al. 2012; Pi et al. 2008). The first three domains of Ccn2/Ctgf protein exhibit a broad binding ability and are required for the interactions with Slit3 (Pi et al. 2012). To verify Ccn2/Ctgf interaction with Slit2, we performed additional yeast two-hybrid analyses. Ccn2/Ctgf and its mutants were fused to GAL4-BD constructs that contained tryptophan gene. The human cDNAs of SLIT2-EGF_7 − 9_, SLIT2-CT, or both were fused to GAL4-AD vectors that expressed leucine gene. All co-transformants that carried BD and AD constructs could grow in the SD agar media lacking tryptophan and leucine (Fig. 4A, right panel). *HIS3* gene was designed in the yeast two-hybrid system to detect any interaction that could bright the GAL4 BD and AD fusion proteins in proximity leading to transcriptional activation of the reporter. We identified interactors that grew in the selection SD agar media containing 3-AT but lacking tryptophan, leucine, and histidine (Fig. 4A, left panel). Using this approach, we confirmed interaction of Ccn2/Ctgf with SLIT2-EGF_7 − 9_ and SLIT2-CT via the first three domain of this protein. To further understand this interaction, we purified MBP fused SLIT2C from *E. coli* strain BL2 (DE3) and found a dose-dependent binding of Ccn2/Ctgf to MBP:SLIT2C in solid-phase assays (Fig. 4B and C). We also co-expressed SLIT2:MYC with Ccn2/Ctgf or its truncated mutant in CHO cells (Fig. 4D). SLIT2:MYC protein was specifically detected in complexes that were immunoprecipitated with M2 antibody and 3xFLAG tagged Ccn2/Ctgf and Ccn2/Ctgf_I,II,III_ whereas it showed little binding to the first two domains of Ccn2/Ctgf (Fig. 4E). The TSP1 alone was not sufficient for the interaction either (data not shown), implicating that the first three domains of Ccn2/Ctgf were required for their binding to SLIT2. Nevertheless, co-localization of murine Slit2 and Ccn2/Ctgf protein was observed in isolated primary HSC that were cultured for a week (Fig. 4F). Exposure of the primary HSC to recombinant murine Slit2 at 200 ng/mL concentration induced PI3K and AKT phosphorylation as well as aSMA upregulation (Fig. 4G). Recombinant murine Ccn2/Ctgf (200 ng/ml) induced PI3K and AKT phosphorylation despite of its little effect on aSMA upregulation (Fig. 4G), indicating that Ccn2/Ctgf alone might induce transient activation of PI3K/AKT signaling that was not strong enough to activate fibrogenic action. Interestingly, this induction of PI3K and AKT phosphorylation as well as aSMA upregulation could be enhanced in presence of the recombinant murine Ccn2/Ctgf and Slit2. In contrast, addition of soluble ROBO1-Fc chimera protein (500 ng/ml), which carries two IgG domains responsible for Slit2 binding and sequestration (Liu et al. 2004), blocked these effects mediated by Slit2 and Ccn2/Ctgf.

### Ectopic Ccn2/Ctgf binds to Slit2 and potentiates murine liver fibrosis

**Fig. 5:**
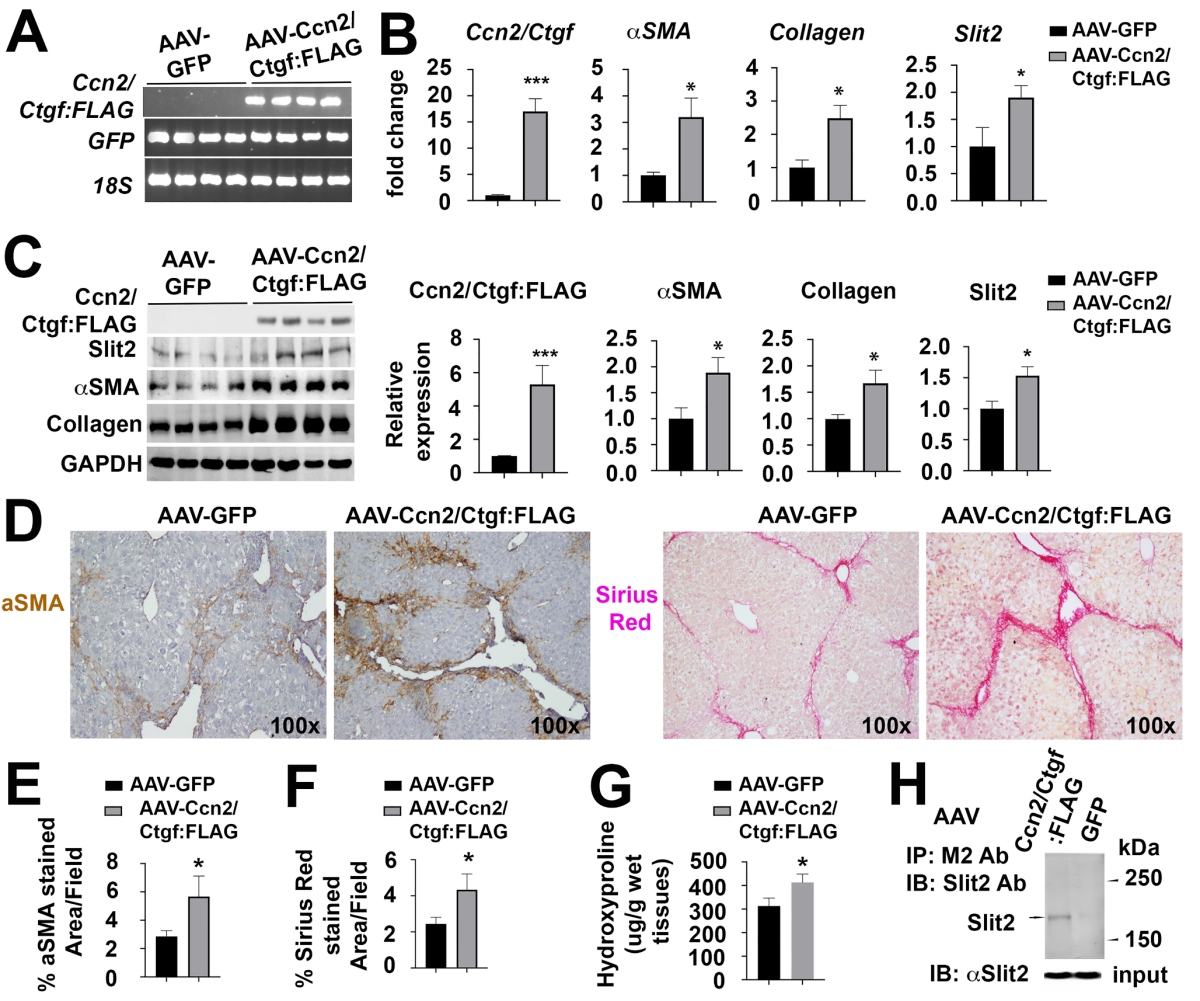
Ccn2/Ctgf binds to Slit2 and potentiates HSC activation and liver fibrosis in vivo. AAV8-Ccn2/Ctgf:FLAG or GFP control was delivered into mice (n = 4 per group). Three weeks later, the mice received 6-week intoxication with CCl_4_. (A) Semi qRT-PCR analysis detected Ccn2/Ctgf:FLAG and co-expressed GFP in infected livers. (B and C) Ectopic expression of Ccn2/Ctgf caused upregulation of *Slit2*, *aSMA*, and *Collagen type I* genes as detected by qRT-PCR (B) and Western analysis (C). The band intensity in (C) was quantified by densitometry in graphs. Data in (B and C) represent means ± SD in relation to AAV8-GFP infected controls (n = 4). *P < 0.05; ***P < 0.001. (D) IHC for aSMA and Sirius red staining. (E and F) Quantification of aSMA^+^ areas and Sirius red stained areas was performed. Values were means ± SEM based on quantification of images from more than 10 fields per mouse (n = 4 mice per group). *P < 0.05. (G) Measurement of hepatic hydroxyproline showed increased Collagen deposition in fibrotic livers that ectopically expressed Ccn2/Ctgf:FLAG. Data are means ± SD (n = 4 mice per group). *P < 0.05. (H) Slit2 and Ccn2/Ctgf:FLAG were pulled down together in total protein lysates isolated from the AAV8-Ccn2/Ctgf:FLAG- or AAV8-GFP-infected livers (upper panel). The M2 antibody conjugated agarose was used for immunoprecipitation and a rabbit Slit2 antibody for immunoblotting. Equal input for cell lysates containing Slit2 protein in the assays were used (lower panel)

To further understand the in vivo function of Ccn2/Ctgf, we used the AAV8 delivery system and ectopically expressed this gene in mouse livers as shown in RT-PCR analysis (Fig. 5A). The recombinant AAV8 virus contained Ccn2/Ctgf:FLAG and IRES-GFP sequences. GFP alone in AAV8 viruses was also expressed in parallel studies. The infected mice were exposed to CCl_4_ for 6 weeks. The qRT-PCR analysis showed higher levels of *Ccn2/Ctgf, Slit2, aSMA*, and *Collagen* in the AAV8-Ccn2/Ctgf:FLAG infected livers than AAV8-GFP controls after the 6-week exposure to CCl_4_ (Fig. 5B). Accordingly, elevated levels of Ccn2/Ctgf, Slit2, aSMA, and Collagen type I proteins were detected in immunoblotting and quantified in densitometry analyses (Fig. 5C). IHC confirmed larger areas of aSMA^+^ myofibroblast cells and Collagen deposition (Fig. 5D-F). Enhanced deposition of Collagen was also detected as indicated by increased hydroxyproline content in the fibrotic livers with ectopically expressed Ccn2/Ctgf (Fig. 5G). Furthermore, the murine Slit2 and Ccn2/Ctgf:FLAG proteins were co-immunoprecipitated using the M2 mouse antibody against FLAG epitope in the AAV8- Ccn2/Ctgf:FLAG infected livers (Fig. 5H). Collectively, these observations indicated that Ccn2/Ctgf formed complexes with Slit2 and its ectopic expression promoted liver fibrosis.

## Discussion

Ccn2/Ctgf is a matricellular protein regulating biological processes ranging from cell proliferation, angiogenesis, wound healing, tumor development, to organ fibrosis. Its germline deletion causes prenatal lethality and multiple skeletal defects in mice (Ivkovic et al. 2003). Conditional deletion of this gene can reduce wound healing responses and tissue fibrosis in multiple organs including lung, cornea, and liver (Gibson et al. 2014; Liu et al. 2011; Pi et al. 2015). Its genetic modulation in muscle is not sufficient to drive fibrosis, but alters Collagen content and organization after injury (Petrosino, Leask, and Accornero 2019). Overexpression of Ccn2/Ctgf protein has been reported in concert with signaling pathways associated with fibrosing injuries for initiation and exacerbation of fibrosis in liver and kidney (Tong et al. 2009; Yokoi et al. 2008). A high level of Ccn2/Ctgf protein renders the livers more susceptible to the injurious actions of other fibrotic stimuli (Tong et al. 2009). In line with central roles of Ccn2/Ctgf in liver fibrosis, we have observed that deletion of exon 4 in this gene in a tamoxifen-inducible manners in floxed mice that carry *ubc-Cre/ERT2* transgene decreases ductular reaction and biliary fibrosis after the feeding of a biliary toxin 3,5-diethoxycarbonyl-1,4-dihydrocollidine (DDC) (Pi et al. 2015). Consistent with these previous findings, this study showed that the conditional *Ccn2/Ctgf* mouse knockouts exhibited reduction in CCl_4_-induced liver fibrosis. Loss of hepatocyte-derived *Ccn2/Ctgf* in rats through AAV8-Cre also attenuated liver fibrosis after chronic intoxication caused by CCl_4_. These observations indicated conserved functions of *Ccn2/Ctgf* in liver fibrosis between mice and rats. On the other hand, rats and mice have differential metabolisms for certain toxins. For examples, 2AAF can inhibit hepatocyte proliferation to induce hepatic progenitor/oval cells in combination with partial hepatectomy in rats (Solt, Medline, and Farber 1977), but not in mice. Given the fact that oval cell studies have been limited due to technical barriers in genetic manipulation and specific cell isolation for rats during past years, our unique model in this species shall help investigate the role of *Ccn2/Ctgf* in rat oval cells that are activated during certain chronic liver injury in future.

Another important finding in this study is about modulation of Slit2 activity through Ccn2/Ctgf interaction. It seems that Ccn2/Ctgf binding to Slit2 protein requires multiple domains including IGFBP1, vWC, and TSP1 repeat. These domains can contribute to the regulation of fibrotic responses through interaction with growth factors such as TGFβ, vascular endothelial cell growth factor, and bone morphogenic proteins leading to enhanced presentations or sequestration of these factors to cognate receptors thereby modulating corresponding downstream signaling (Abreu et al. 2002), (Inoki et al. 2002). Thus, Ccn2/Ctgf binding may directly influence bioactivity, bioavailability, and presentation of Slit2 protein to Robo1 receptor. Moreover, Ccn2/Ctgf protein may crosstalk with Slit2/Robo signaling due to its adhesive abilities with integrins. For instances, the TSP1 repeat interacts with integrin a6b1 and stimulate Collagen deposition (Heng et al. 2006). The IGFBP1 domain binds to integrin a5b1 and stimulates rat oval cell adhesion (Pi et al. 2008). Although the C-terminal CT domain in Ccn2/Ctgf protein is not necessary for Slit2 interaction, it can directly interact with integrins such as a5b1 and avb3 (Tong and Brigstock 2006; Gao and Brigstock 2004). Integrins a5b1 and avb3 can interact with adhesive ligands through recognition of an Arg-Gly-Asp (RGD) binding motif. To our surprise, Robo1 receptor contains a RGD site in many species including human (Genbank accession# NM_133631.4), mice (Genbank accession# NM_019413), and rats (Genbank accession# NM_022188). It is easy to speculate a direct association among Ccn2/Ctgf, integrins, Slit2, and Robo1 as a whole complex on HSC cell surface during liver fibrosis. Integrins is cellular receptors for Ccn2/Ctgf and regulates a myriad of cellular activities including cell adhesion, migration, proliferation, and survival. Potential integrin binding to Robo1 via its RGD site on HSC cell surface may synergically promote downstream intracellular signaling.

Emerging evidence have demonstrated Slit2 involvement in multiple types of liver pathologies. Slit2-Robo1 signaling promotes intrahepatic angiogenesis during ductular reaction (Coll et al. 2022). Slit2 signaling contributes to cholestatic fibrosis in mice by activation of HSC (Li et al. 2019). SLIT2 has also been identified as a driver of tumor dissemination and tumor-associated neutrophil infiltration in relapsed human intrahepatic cholangiocarcinoma (Zhou et al. 2021). Moreover, ROBO1 and SLIT2 are induced in HCC and neighboring cells and shed into serum in humans (Ito et al. 2006). This pair of receptor and ligand can differentiate histopathological subgroups of liver tissues depending on both tumor staging and differentiation status (Avci, Konu, and Yagci 2008). Coincidently, Ccn2/Ctgf has been found to mediate tumor-stroma interaction between hepatoma cells and HSC to accelerate HCC progression (Makino et al. 2018). It is conceivable that the interactions of Slit2 and Ccn2/Ctgf activate Robo and integrin resulting in promoted HSC activation and HCC development in vivo. In supporting of this concept, crosstalk between the activated Slit2-Robo1 pathway and TGFβ1 signaling has been found to promote cardiac fibrosis (Liu et al. 2021). Inhibiting crosstalk of the two profibrotic pathways by targeting Ccn2/Ctgf and Slit2 interaction may help develop therapeutic strategies against HSC activation and liver scaring in chronic liver disease.

In summary, this paper presented a novel interaction between Ccn2/Ctgf and Slit2 during liver fibrosis. The two ligands may work in concert to stimulate their corresponding receptors-integrins and Robo1 as part of regulatory mechanisms in their common functions, including inflammatory cell recruitment, angiogenesis, and fibrosis during tissue damage. Further investigations are warranted to examine synergistic actions in integrin and Robo1 signalings after Ccn2/Ctgf and Slit2 stimulation in liver cells such as HSC and vascular endothelial cells during inflammation, angiogenesis, and fibrosis after hepatic damage.

## Electronic supplementary material

Below is the link to the electronic supplementary material.

Supplementary Material 1

## Data Availability

All data supporting the findings of this study are available within the paper and within its supplementary materials published online.
