# Supplementary material for: CCN2/CTGF promotes liver fibrosis through crosstalk with the Slit2/Robo signaling
Source: J Cell Commun Signal. 2022 Dec 5;17(1):137–50. doi: 10.1007/s12079-022-00713-y (PMC10030765; doi:10.1007/s12079-022-00713-y)
Supplement: Supplementary file 1 — Supplementary Material 1 [file 12079_2022_713_MOESM1_ESM.docx]

### CCN2/CTGF Promotes Liver Fibrosis Through Crosstalk with the Slit2/Robo Signaling

**Authors:** Liya Pi^1#^, Chunbao Sun^1^, Natacha Jn-Simon^1^, Sreenivasulu Basha^1^, Haven Thomas^1^, Victoria Figueroa^1^, Ali Zarrinpar^3^, Qi Cao^2^, Bryon Petersen^4^

**Affiliations:**

^1^Department of Pathology, Tulane University, New Orleans, LA, USA

^2^Department of Diagnostic Radiology and Nuclear Medicine, University of Maryland School of Medicine, Baltimore, MD, USA

^3^Department of Surgery, ^4^Department of Pediatrics, University of Florida, Gainesville, FL, USA

^#^ **Correspondence author**

Liya Pi, PhD

Department of Pathology

Tulane University School of Medicine

1430 Tulane Ave

New Orleans, LA, USA

E-mail: lpi@tulane.edu

Phone: (504)-988-2869

**Conflict of Interests**

The authors declare that there is no conflict of interest regarding publication of this manuscript.

**Financial Support**

This study is supported by National Institutes of Health NIAAA **R01AA028035** grant.

**Supplemental Figure Legend**

**Supplemental Figure 1.** **Generation of floxed homozygotes for Cre-mediated deletion of *Ccn2/Ctgf* gene in rats.** (A) A diagram shows a strategy using the CRISPR technology to generate a floxed targeted allele that contains two loxP sites flanking *Ccn2/Ctgf* loci (*Ccn2/Ctgf^flox/flox^*) for Cre-mediated deletion for conditional knockout in rats. A donor vector was constructed by in-fusion technology to contain a 5’ homologous arm (3.0 kb), a flox region (2.8 kb), and a 3’ homologous arm (3.0 kb). The mixtures of Cas9 mRNA gRNAs and donor vector were microinjected into fertilized eggs of SD rats. (B) PCR analyses using I and II primer pair (indicated by red arrowheads) (5’ GAACAAGTCCAGATAAACGCAGAG 3’ and 5’ CAGCTTCCCTTGATGGTAG 3’) or III and IV primer pair (green arrowheads) (5’ GGGCACTCTCCTTAAACTCCATC 3’ and 5’ TGTGTCTGCCCCATCATTCC 3’) detected 6.5-kb PCR products for the 5’ (left panel) or 3’ homologous arms (right panel) respectively in 7 F1 rats that had homologous recombination. In contrast, wild-type PCR products were about 9.5 or 9.6 kb for 5’ or 3’ homologous arm respectively. (C) The PCR-based genotyping revealed that the floxed allele gave rise to a PCR product about 271 bp whereas the wild type of PCR product about 204 bp using P1 and P2 primer pair (indicated by black arrowheads) 5’ CCATGCCCAGTCATTGTCCT 3’ and 5’ CCGCCCAAAATGCCTATGTG 3’. (D) RT-PCR analysis showed that the floxed homozygotes that received AAV8-GFP had a 304-bp PCR product corresponding to exons 2 and 3 of *Ccn2/Ctgf* gene using RT1 and RT2 primers [indicated by light red arrowheads; 5’ ATCCCTGCGACCCACACAAG 3’ (RT1) and 5’ CAACTGCTTTGGAAGGACTCGC 3’ (RT2)]. In contrast, the conditional knockouts that received AAV8-TBG-iCre barely detected this *Ccn2/Ctgf* fragment. *18S* was used as loading controls.


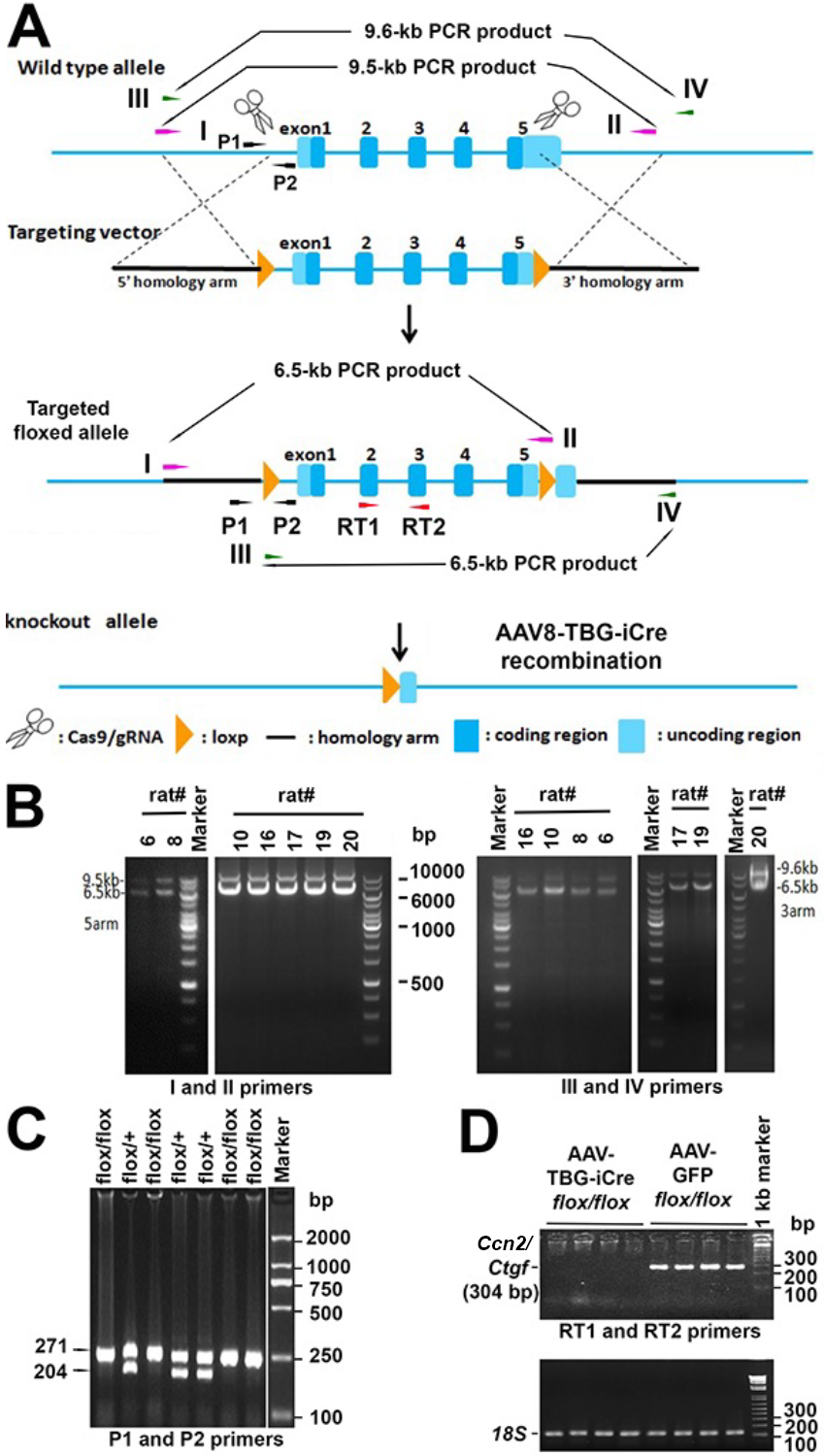


**Supplemental Figure 2. The immunofluorescent staining for Albumin on isolated primary rat *Ccn2/Ctgf^flox/flox^*** **hepatocytes.** *Ccn2/Ctgf^flox/flox^* rats (8-week-old age) were subjected to CCl_4_ intoxication (0.5 μl/g body weight, twice a week for 6 weeks). Two days after the last CCl_4_ injection, the rat livers were perfused through two-step collagenase digestion. Hepatocytes were isolated after centrifuge at 50x and the dead debris was removed after centrifuge with 45% Percoll. The isolated cells were seeded in collagen coated plates and cultured in 37^o^C incubator with 5% CO2 in William medium E for 4 hours. Then the cells were fixed in 4% paraformaldehyde before immunostaining with a rabbit anti-Albumin antibody. DAPI was used to stain nuclei. Magnification: 200x.


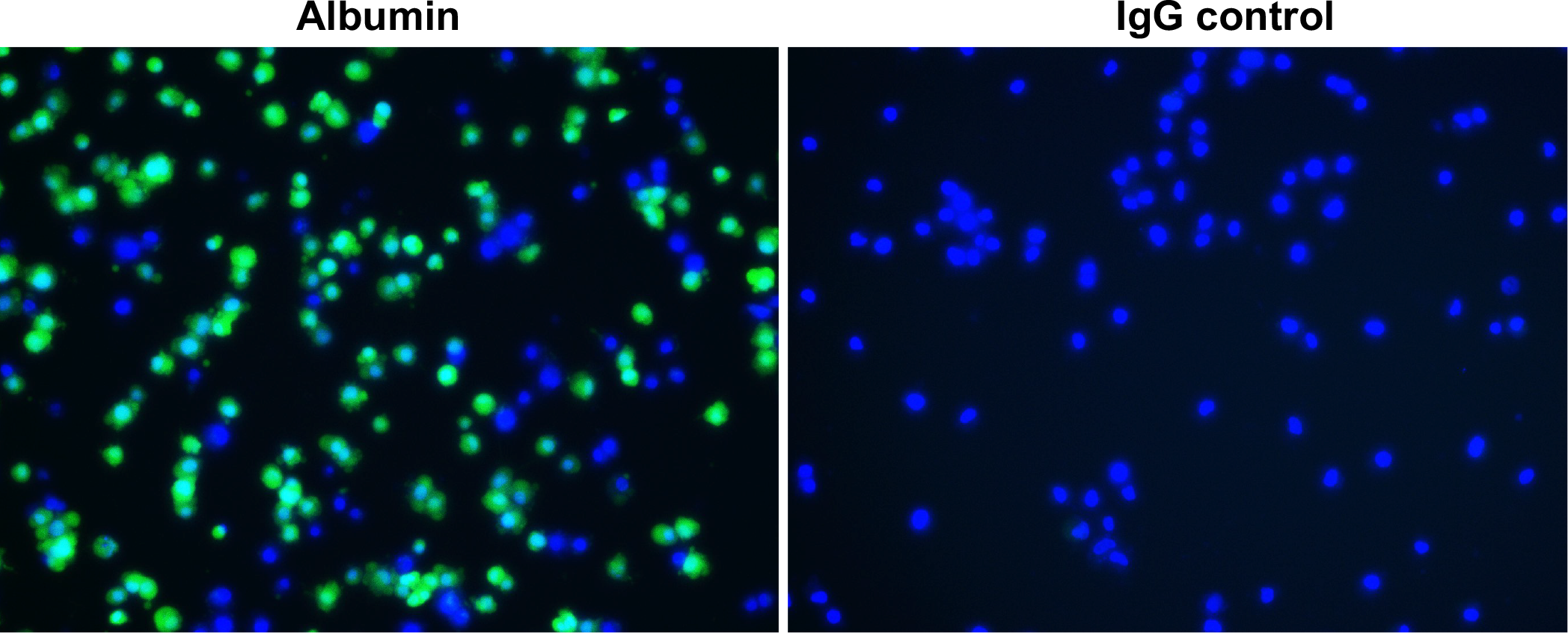


**Supplemental Figure 3.** **GFP visualization shows high efficiency of AAV8-GFP transduction in primary hepatocytes isolated from *Ccn2/Ctgf^flox/flox^* rats.** Animals (8-week-old age) received CCl_4_ intoxication (0.5 μl/g body weight, twice a week for 6 weeks) via IP injection. Two days after the last injection, hepatocytes were perfused through two-step collagenase digestion and cultured in 37^o^C incubator with 5% CO2 in William medium E for 4 hours followed by transduction with AAV8-GFP at a MOI of 100,000 viral genomes/cell. GFP was visualized 4 days post transduction. Nuclei was stained with DAPI. Scale bar: 15 μm.


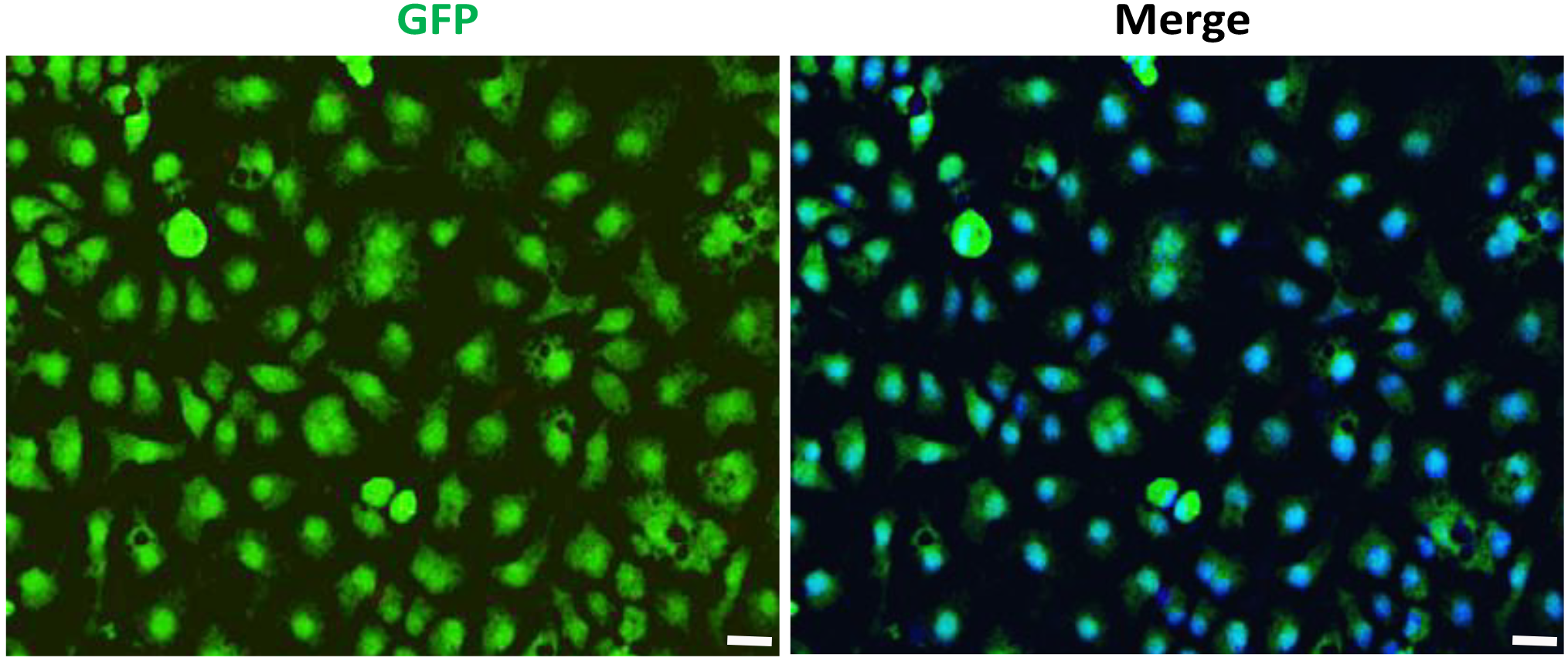


**Supplemental Figure 4.** **Little expression of Ccn2/Ctgf protein in normal adult mouse livers.** Representative images were stained by a rabbit Ccn2/Ctgf antibody and DAPI for nuclei from animals at 8-week-old age (n=3) without CCl_4_ administration. Magnification: 200x.


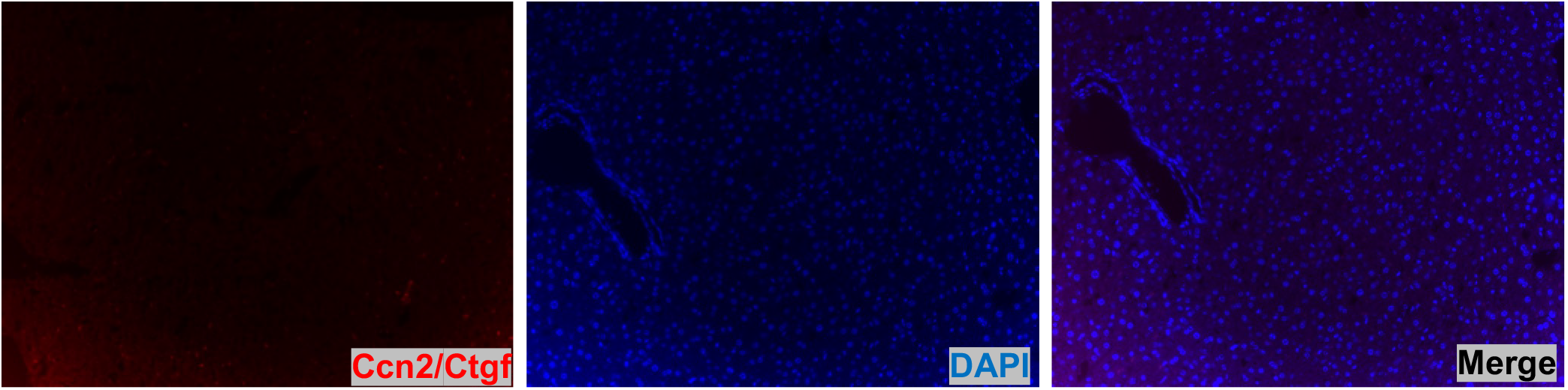


**Supplemental Figure 5. Ccn2/Ctgf** **was induced in ballooning hepatocytes (indicated by white arrow) that were closely associated with αSMA^+^ activated HSC during liver fibrosis.** Dual staining for Ccn2/Ctgf (green signal) and αSMA (red signal) were performed on rat fibrotic livers (6 weeks post CCl_4_) (n=3 mice). Magnification: 200x.

**
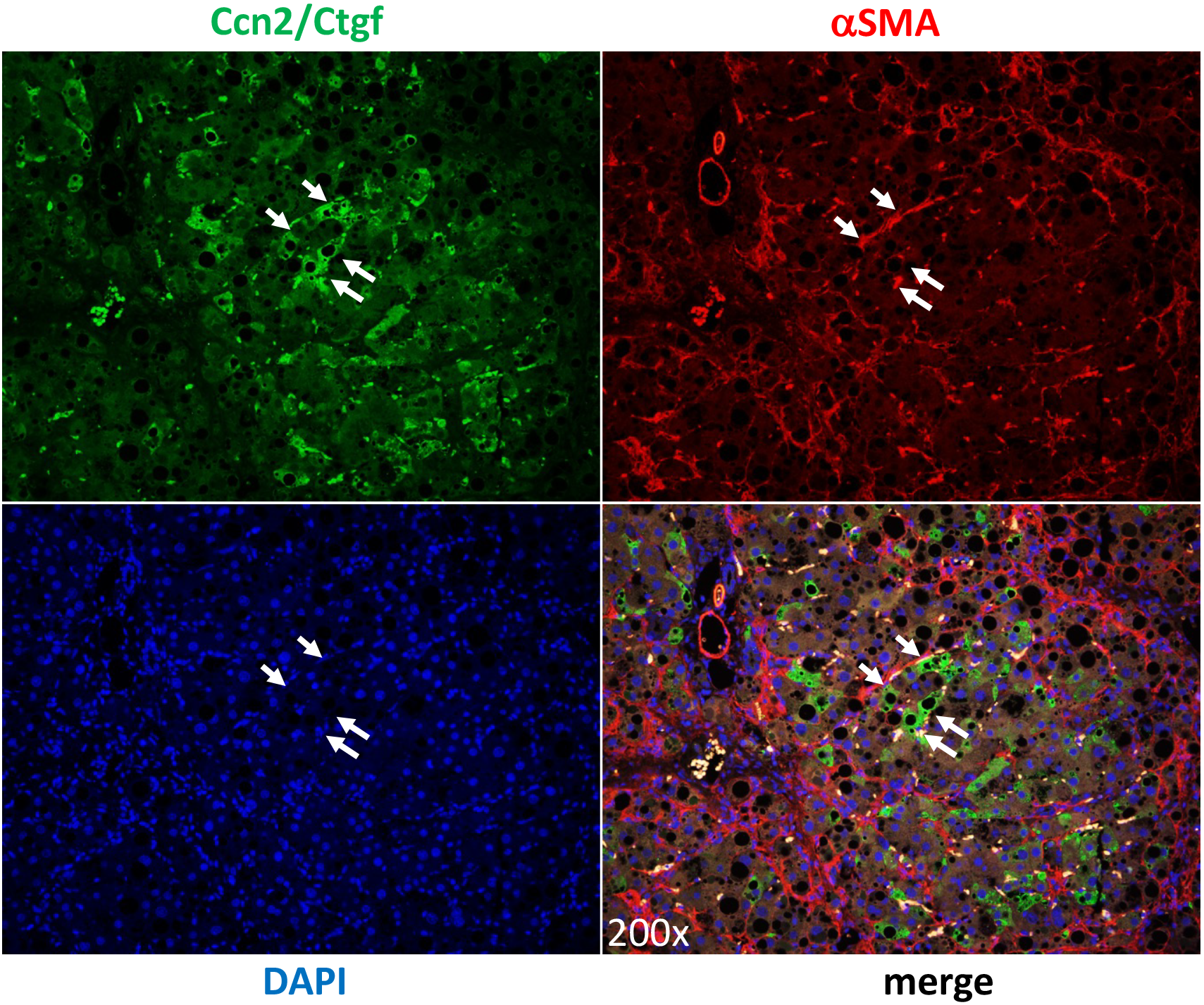
**
